# The Effect of Light Intensity, Strain, and Age on the Behavior, Jumping Frequency and Success, and Welfare of Egg-Strain Pullets Reared in Perchery Systems

**DOI:** 10.3390/ani11123353

**Published:** 2021-11-24

**Authors:** Jo Ann Chew, Tina Widowski, Eugenia Herwig, Tory Shynkaruk, Karen Schwean-Lardner

**Affiliations:** 1 Department of Animal and Poultry Science, University of Saskatchewan, Saskatoon, SK S7N 5A8, Canada; joann.chew@usask.ca (J.A.C.); herwig.eugenia@usask.ca (E.H.); tory.shynkaruk@usask.ca (T.S.); 2 Department of Animal Biosciences, University of Guelph, Guelph, ON N1G 2W1, Canada; twidowsk@uoguelph.ca

**Keywords:** Lohmann Brown-Lite, Lohmann LSL-Lite, novel object test, heterophil/lymphocyte ratio, environmental navigation

## Abstract

**Simple Summary:**

The effect of light intensity on pullet behavior and welfare is not well studied. In this study, two strains (Lohmann LSL-Lite and Lohmann Brown-Lite) of pullets reared in floor pens containing a perchery system were tested under one of three light intensities (10, 30, or 50 lux). Behavior, jumping frequency and success, fear, and stress levels were assessed throughout the study. Pullets reared at 50 lux spent more time preening (comfort behavior) than pullets reared at 10 lux, while pullets reared at 10 lux spent more time pecking at walls (exploratory behavior). All pullets increased their time spent preening with age. The number and accuracy of jumps also increased with age. Light intensity did not affect landing success, nor did it affect pullet fear or stress levels. Lohmann-LSL-Lite pullets performed more jumps than Lohmann Brown-Lite pullets, while Lohmann Brown-Lite pullets spent more time performing exploratory behaviors. Lohmann Brown-Lite pullets also scored higher on the fear and stress assessments, which might suggest genetic differences between the two strains. Overall, the results suggest that light intensity does not affect pullet behavior, although higher light intensity at 50 lux may slightly increase preening in the pullets, which may indicate positive welfare attributes.

**Abstract:**

The effects of light intensity (L) are not well studied in pullets. Our research objective was to study the effect of L on navigational success, behavior, and welfare of two pullet strains (S). In two repeated trials, a 3 × 2 × 4 factorial arrangement tested three L (10, 30, 50 lux) and two S (Lohmann Brown-Lite (LB), LSL-Lite (LW)) at four ages. One thousand eight hundred pullets/S (0–16 wk) were randomly assigned to floor pens within light-tight rooms (three pens/S/room, four rooms/L) containing four parallel perches and a ramp. Data collection included jumping frequency and success (24h continuous sampling), novel object tests (fear), heterophil to lymphocyte (H/L) ratios (stress), and behavior (instantaneous scan sampling) during photoperiods. L did not affect injurious behavior, fear, or H/L. Pullets reared at 50 lux spent more time preening than at 10 lux. Pullets reared at 10 lux spent more time wall pecking than at 50 lux. Time spent standing and preening and total number and accuracy of jumping increased with age. Pullets reared at 30 lux had higher jumping frequency than at 10 lux; accuracy was not affected. LW jumped more than LB, but with similar success. LB spent more time exploring and scored higher in the fear and stress assessments, suggesting S differences.

## 1. Introduction

Studies showed that laying hens are more successful at using complex housing environments when reared in a similar type of housing, as it allows learning to occur early in life [1]. Light intensity (L) may play a role in helping pullets navigate these complex environments by increasing visual acuity. Light intensity may also have an impact on bird behavior and welfare. Many studies have been conducted on L on broilers, while only a few looked at hen behavior and jumping ability [2,3]. Further, information on the impact of L levels on pullets is not well known.

Several variables might affect a pullet’s ability to successfully navigate an environment. Increasing L may result in better navigational ability, as a brighter environment may improve poultry vision [2,4]. Previous studies on laying hens reported increased bird activity and jumping success in a brighter environment [5,6]. Age could be another factor, as Kozak et al. [7] reported that pullets at 10–16 wk performed more aerial ascents than hens at 17–24 and 25–37 wk. It is possible that jumping behavior may decrease with age as younger animals have higher levels of energetic capacity and movements than older animals [8]. Therefore, results from hen studies cannot be directly applied to pullets. Bird strain (S) may also play a role in pullet behavioral and navigational qualities [9,10,11], and should be considered when evaluating the effect of L on pullets.

While increasing L may have positive impacts on birds, it may also result in increased injurious pecking and cannibalism [3,12,13]. Increased injurious behavior within the flock can impact fear and stress levels. Heterophil/lymphocyte (H/L) ratios are considered to be a reliable measure for chronic stress [14]; however, the effect of L on hen or pullet H/L ratios has not been well-studied, as most research focused on hen jumping ability at lower L settings. Information on fear levels in hens in relation to L is also not well-known; however, one study by Hughes and Black [15] reported that hens were more fearful in 17–22 lux than in 55–80 lux.

The present study was conducted to determine whether increasing L can aid pullets in navigating a complex environment without negative behavioral and welfare consequences. The objectives of this study were to examine the effects of L on the behavior, jumping frequency and success, fear, and stress of Lohmann Brown-Lite (LB) and Lohmann LSL-Lite (LW) pullets reared to 16 wk. Three light intensities were tested: 10 (current industry recommended value), 30, and 50 lux. The following hypotheses were tested: (1) L higher than 10 lux would increase active behavior, as well as jumping frequency and success, due to increased visual acuity and bird activity; (2) L higher than 10 lux would increase stress levels, which will increase injurious behavior, fear levels, and H/L ratio; (3) differences between S would result in different measured outputs; and (4) pullet behavior and jumping frequency and success would increase with age as pullets learned to navigate their surroundings.

## 2. Materials and Methods

### 2.1. Animal Housing and Husbandry

The effect of L during pullet rearing on use of space, behavior, fear, and stress were evaluated over two 16 wk blocked trials. Three L, 10, 30, and 50 lux, were evaluated on six individually controlled, light-tight rooms. In each trial, LB and LW pullets (*n* = 900 per S), obtained from a commercial hatchery (Clark’s Poultry, Brandon, MB, Canada), were reared from 0 to 16 wk of age. Pullets were randomly assigned to a pen within a room (50 pullets per pen, six pens per room), and each room was randomly assigned to one of the three L treatments (*n* = 300 pullets per L × S). The average stocking density achieved was 6.5 birds/m^2^, in accordance with the recommendations in the Lohmann Management Guide [16]. Each pen (4.0 m × 2.3 m) was bedded with 7–10 cm depth of wheat straw and furnished with a perching system, ramp, two pan feeders, and a drinker line with six nipples (Lubing Systems LP, Cleveland, TN, USA). The perching system (height 0.56 m × width 1.16 m × length 2.18 m) consisted of four wooden rectangle perches (length 3.8 cm × height 3.5 cm) spaced 30 cm apart with the top corners angled to allow for easy grasping. The ramp (length 81.3 cm × width 48.3 cm at an angle of 38°) was made of 14-gauge wire with 2.54 cm × 2.54 cm dimensions. The ramps were added to the perching system at 14 d to prevent pullets’ toes from becoming trapped in the ramp wires prior to that time. The pan feeders used initially had a 36 cm diameter and 113 cm circumference and were replaced with larger pans (44 cm diameter and 138 cm circumference) at 6 wk of age. All birds had ad libitum access to water and commercial feed appropriate for their stage of development [16] and also had access to supplemental feeders and waterers in the first week. The pullets were vaccinated for Marek’s Rispens, HVT-IBD, and Poulvac ST at the hatchery. They were also vaccinated for Newcastle bronchitis at the ages of 2, 6, and 10 wk, *Salmonella typhimurium* at 9 and 11 wk, and Newcastle bronchitis and *Salmonella enteriditis* at 15 wk. Birds were checked a minimum of twice daily throughout the trial.

Lighting was provided via eight 11-watt white-light-emitting diode (LED) light bulbs (2821 Kelvin, Greengage Agritech Limited, Roslin Innovation Centre, Midlothian, UK) per room. The light bulbs were positioned so L was similar in all pens when measured at bird level in the center of the pen. The pullets were provided with a photoperiod of 23L:1D for the first week, which gradually decreased until 7 wk of age, where the photoperiod remained at 8L:16D until the end of trial [17]. For the first week, L was set at 50 lux for all rooms to ensure all chicks were able to easily locate feed and water. The L settings were adjusted according to the assigned room-appropriate intensity treatment after the first week. Light intensity was measured with a lux meter every 2 wk (Extech LT300, Extech Instruments, Montreal, QC, Canada), and any variances corrected back to planned intensity. Dawn and dusk periods were simulated over two 15 min periods daily. Room temperature was set at 33 °C on the first day; heat was provided via hot water pipes running along the walls of the rooms. Room temperature was gradually decreased daily until 20 °C at week 5, where it was maintained in accordance with industry recommendations [16]. All rooms were ventilated via a negative pressure inlet-fan system.

### 2.2. Data Collection

#### 2.2.1. Behavior

Pullet behavior was recorded on a pen basis (per S per room) with infrared cameras (Panasonic WV-CF224FX, Panasonic Corporation of North America, Secaucus, NJ, USA) for 24 h periods at 4, 8, 13, and 16 wk of age. The cameras captured the entire area of the pen. Videos were analyzed with the Genetec Omnicast software (Genetec Inc., Montreal, QC, Canada). Instantaneous scan sampling was conducted at 20 min intervals during the light period (the length of the photoperiod was 12 h at 4 wk, 8 h at 8, 13, and 16 wk), according to the ethogram presented in Table 1.

To determine bird navigation between pen furnishings, the 24 h recordings were also used to conduct continuous behavior sampling. Jumps and flights of both takeoff and landing locations were recorded, as well as whether the landing was a success or failure. Success was determined when a pullet jumped from one part of the pen environment or equipment to another and reached or landed in its target location without incident (falling or crashing), while failure was classified as when the pullet did not reach its targeted location, and instead crashed into it or fell. Pen jumping and landing locations included perches, ramps, drinker lines, top of feeder bins, and the floor. Jumping and landing success were also determined as a percentage based on the number of successful and failed jumps over the total number of jumps performed. Post observations, these jumps were categorized into jumps upward, downward, or across. Several jumps were too infrequent to justify analysis, including jumps from the perch to the top of the feeder bin, from the perch to the ramp and drinker line, from the ramp to the perch, floor, drinker line, or top of feeder bin, from the floor to the top of the feeder bin, from the drinker to the perch and top of feeder bin, and from the top of the feeder bin to the perch, floor, or drinker line.

One observer conducted the observations for both instantaneous scan sampling and continuous behavior sampling. The observer was blind to L treatments, but not to S treatments, since it was easy to tell between S in the video recordings. However, it was not possible to tell between L treatments; therefore, the observer was blind to L treatments. Prior to beginning observations, inter-observer reliability was tested by having a second observer watch the same footage (10 footages per strain at 4 wk), calculating the percent agreement for each behavior, and obtaining an average minimum of 80% consistency across data.

#### 2.2.2. Novel Object Test

At 15 wk of age, pullet fear responses were assessed using a novel object test. A foil tie-dyed balloon weight (Unique 4927, Fancy Dress Worldwide, Worcester, UK) was placed on the pen floor, approximately 0.6 m from the pen entrance. Pullets housed in two pens per S per room were evaluated by recording the latency for three separate birds to peck at the novel object with a maximum allotted time of 900 s (15 min per observation). All pens in a room were tested by live observation at the same time with four different testers randomly assigned to each pen and with each pen observed individually. Tests began at 8 a.m. and were concluded at 9:30 a.m. An average latency to peck at the object for all three pecking times was recorded in seconds and used for analysis.

#### 2.2.3. H/L Ratio

To assess chronic stress, blood was collected from two birds per pen per room at 15 wk of age for analysis of H/L ratio. Using a 22-gauge needle, 2 ml of blood were collected from the brachial vein in an ethylenediaminetetraacetic acid (EDTA) anti-coagulation vacutainer. Within 30 min of collection, the blood from each bird was used to create two duplicate smear stains. After drying for 24 h, the slides were stained using PROTOCOL™ Hema 3™ (Fisher Scientific, Ottawa, ON, Canada). The slides were then read using a light microscope (Optika© B-290TB, Bergamo, Italy) fitted with 100× field of view with oil magnification. Up to 100 heterophil or lymphocyte cells were counted, and the H/L ratio was determined by dividing the number of heterophils by the number of lymphocytes. One observer conducted the observation and was blind to both L and S treatments. Prior to beginning observations, inter-observer reliability was tested by having a second observer watch the same field of view, calculating the percent agreement for each field of view, and obtaining a minimum of 80% consistency across data.

### 2.3. Statistical Analyses

The experiment was designed as a 3 L × 2 S × 4 wk factorial arrangement within a randomized complete block design. Trial was treated as a block. Room was nested within L and was also the replicate unit for L (two repetitions per L treatments per trial). Pen was the replicate unit for S (three replicates per S per room per trial). All data were checked for normality using the UNIVARIATE Procedure in SAS 9.4^®^ (SAS^®^ 9.4, Cary, NC, USA), and any data not meeting normality assumptions were log transformed (data log+1) prior to analyses. An analysis of variance (ANOVA) test was done using the MIXED Procedure (SAS^®^ 9.4, Cary, NC, USA) to determine differences among group means. Behavior and jumping frequency and success were analyzed as a two-way repeated measure ANOVA. For all data, a Tukey’s range test was used to separate means. For all statistical analyses, significance was declared when *p* < 0.05 and trends noted at 0.05 ≤ *p* < 0.10.

## 3. Results

### 3.1. Behavior

The effects of L, S, and week on pullet behavior is reported in Table 2. There was no interaction between L, S, and week. There was an interaction between L and week on time spent jumping or flying. At 4 wk, pullets reared at 10 or 30 lux spent more time jumping or flying (0.11% and 0.12%) than pullets reared at 50 lux (0.04%, *p* = 0.02, Table 3). Time spent jumping or flying decreased with increasing week for pullets reared at 30 lux, while it remained constant for pullets reared at 10 lux. Pullets reared at 50 lux spent the most time jumping and flying at 8 wk (0.42%, Table 3).

There was also an interaction between L and week for time spent pecking at objects in the environment (Table 2). These included perches, ramps, feeder bins (without consumption), and drinker lines (without consumption). Pullets reared at 50 lux spent the least amount of time pecking at objects in the environment at 4 wk (Table 3). Overall, in all L treatments, time spent object pecking increased with week.

An interaction between S and week was also observed for object pecking. In fact, the two S expressed exploratory behaviors differently with week. Exploratory behaviors included gentle pecking, litter-directed pecking, wall pecking, and object pecking. Time spent gentle pecking increased in LB pullets with age (0.20% at 4 wk, 0.18% at 8wk, 0.37% at 13 wk, 0.77% at 16 wk), while for LW pullets, time spent gentle pecking peaked at 8 and 13 wk (0.65% and 0.47% vs. 0.22% at 4 wk and 0.16% at 16 wk, *p* < 0.05, Table 3). Time spent litter-directed pecking was higher in LB pullets than LW pullets at 4 (23.04% vs. 16.31%, *p* < 0.05), 8 (20.97% vs. 13.83%, *p* < 0.05), and 13 wk (18.58% vs. 15.06%, *p* < 0.05). Within strain, time spent litter-directed pecking decreased with age for LB pullets, and there was no S effect on time spent litter-directed pecking for LW pullets. For wall pecking, LB pullets spent more time wall pecking at 13 and 16 wk (4.43% and 5.61% vs. 1.41% at 4 wk and 3.11% at 8 wk), while LW pullets spent similar amounts of time performing this behavior throughout all recorded observations (1.99%, 2.32%, 2.22%, and 2.34% at 4, 8, 13, and 16 wk, respectively, *p* > 0.05). Both S increased the time spent object pecking with week. LW pullets spent more time object pecking at 8 (0.46%), 13 (0.60%), and 16 wk (0.66%) than 4 wk (0.16%), while LB pullets spent the most time object pecking at 16 wk (1.11% vs. 0.26%, 0.26%, and 0.39% at 4, 8, and 13 wk, respectively, Table 3).

There was an interaction between S and week for time spent at the feeder. Both S decreased time spent at the feeder at 16 wk (7.22% vs. 10.60% at 4 wk for LB, *p* < 0.05, 9.20% vs. 12.24% at 4 wk for LW, *p* < 0.05). There was also an interaction between L and S for the percentage of unidentified behaviors, where LB pullets’ behaviors were consistently more difficult to identify than LW pullets at all L treatments (Table 3).

There was an effect of L on preening and wall-pecking behavior. Pullets reared at 30 (11.3%) and 50 lux (12.1%) spent more time preening than pullets reared at 10 lux (10.1%, *p* < 0.05, Table 2), while pullets reared at 10 lux spent more time pecking at walls (3.8%) than pullets reared at 30 (2.7%) or 50 lux (2.3%, *p* < 0.05, Table 2).

For the effect of S on pullet behavior, LW pullets spent more time resting (17.0%) and preening (12.6%) than LB pullets (10.8% and 9.8%), while LB pullets spent more time performing litter-directed pecking (19.7%) and wall pecking (3.6%) than LW pullets (15.2% and 2.2%).

All pullets increased the time spent preening with age. Standing was highest at 16 wk (32.5%), followed by 13 wk (25.5%), 4 wk (20.6%), and 8 wk (15.2%). Time spent walking was lowest at 13 wk (4.0%) compared to all other recorded periods (5.3%, 5.4%, and 4.9% at 4, 8, and 16 wk, respectively, *p* < 0.05, Table 2). Time spent resting was highest at 8 wk (21.0%), followed by 13 (14.5%), 4 (11.4%), and 16 wk (8.7%). Time spent performing other comfort behaviors was higher at 8 (1.6%) and 13 wk (1.5% vs. 0.9% and 0.7% at 4 and 16 wk, respectively, *p* < 0.05, Table 2). Time spent at the drinker was highest at 8 wk (4.0% vs. 3.0%, 3.4%, and 3.0% at 4, 13, and 16 wk, respectively, *p* < 0.05, Table 2). Altogether, pullets’ behaviors were easier to identify at 13 and 16 wk (7.5% and 8.3% unidentified behavior) than at 4 (19.7%) or 8 (10.1%) wk. Finally, neither L, S, nor week affected injurious behavior in pullets.

### 3.2. Jumping Frequency and Success Rate

There were several interactions between S and week (Table 4). For jumps directed upward from the floor to the drinker, LW pullets had the highest number of successful jumps at 4 wk (average 7.75 jumps per pullet over 24 h vs. 1.62 jumps, Table 5). LW pullets also had the highest number of failed landings from the floor to the drinker at 4 wk (0.17 jumps vs. 0.03, 0.02, 0.01, and <0.01, *p* < 0.05, Table 5). There was no interaction between S and week on percent success of jumps from the floor to the drinker (Table 4). This same relationship was also observed for jumps from the floor to the ramp; LW pullets performed both more successful and failed jumps at 4 wk (1.77 and 0.03) than LB pullets (0.78 and 0.01) (Table 5). However, percent success of landing from the floor to the ramp was unaffected by the interaction between S and week (Table 4). Another type of jump directed upward was from the floor to the perch. At all recorded weeks, LW pullets performed more successful jumps from the floor to the perch than LB pullets (8.59, 15.22, 15.00, and 15.16 vs. 1.15, 5.49, 6.78, and 6.25 at 4, 8, 13, and 16 wk, respectively, *p* < 0.05, Table 5). Within S, overall, the number of successful jumps and jumping accuracy increased with week (Table 5).

For jumps directed downward, LW pullets successfully jumped from the drinker to the floor the most at 4 wk (7.62 vs. 3.01, 3.46, 3.84 at 8, 13, and 16 wk, respectively), while that specific behavior in LB pullets peaked at 8 wk (2.94 vs. 1.57, 2.37, and 2.55 at 4, 13, and 16 wk, respectively, Table 5). LB pullets had a higher number of failed landings from the drinker to the floor at 4 wk; however, the difference was numerically minute (0.01 at 4 wk vs. 0.00 at 8, 13, and 16 wk), and total jumping accuracy was unaffected. On the other hand, jumping accuracy was affected for jumps from the perch to the floor; at 4 wk, LB pullets had a lower percent success than the other weeks. Despite this, the difference in percent success was numerically minute (99.77% vs. 100% at 8, 13, and 16 wk, *p* < 0.05, Table 5). Percent success for LW pullets from the perch to the floor was similar across ages.

There was also an interaction between L and S for failed jumps from the perch to the floor. LW pullets reared at 10 lux had a higher number of failed landings (0.01) than LB pullets (0.00). However, differences were negligible.

For jumps between perches, LW pullets performed the most successful jumps at 4 (8.02) and 8 wk (9.97). LW pullets performed fewer successful jumps at 13 (5.92) and 16 wk (5.77) compared to LB pullets (7.79 and 8.04 at 13 and 16 wk, respectively), who peaked in jumps between perches at 8 wk (8.98).

Overall, for differences between S jumps across weeks, LW pullets had more total successful jumps than LB pullets (43.94 vs. 14.40 at 4 wk, 43.66 vs. 24.29 at 8 wk, 41.03 vs. 23.46 at 13 wk, and 42.41 vs. 23.86 at 16 wk), while within S, LB pullets had more total successful jumps at 8, 13, and 16 wk than 4 wk (*p* < 0.05, Table 5). LW pullets also had the most failed landings between S and across all weeks (0.33 vs. 0.12 at 4 wk, 0.10 vs. 0.05 at 8 wk, 0.04 vs. 0.02 at 13 wk, and 0.04 vs. 0.02 at 16 wk). Both S performed fewer failed landings with increasing week; however, total percent success was not affected between S and week.

L impacted pullets jumping between perches. Pullets reared at 30 lux had a higher number of successful jumps (8.3) than pullets reared at 10 lux (6.9), with pullets reared at 50 lux showing an intermediate response (7.6, Table 4). Despite the higher jumping frequency, jumping accuracy was not affected by L.

S impacted the frequency and success of several different jumps. For jumps directed upward from the floor to the perch, LW pullets had higher failed landings than LB pullets (<0.1 vs. <0.1, respectively, *p* < 0.01, Table 4). For jumps directed downward from the drinker to the floor, LB pullets had a higher jumping accuracy (100.00%) than LW pullets (99.97%). LW pullets had a higher number of successful jumps from the perch to the floor (11.8) than LB pullets (3.5, *p* < 0.01).

Finally, age had several effects on jumping frequency and success of pullets. Jumping accuracy increased with age for jumps from the floor to the drinker (97.83%, 98.77%, 99.66%, 99.85% at 4, 8, 13, and 16 wk, respectively, Table 4). Failed landings from the floor to the perch decreased with age (0.1 at 4 wk, <0.1 at 8, 13, and 16 wk). The number of jumps from the ramp to the floor decreased with age (0.4 at 4 wk vs. 0.1 at 8, 13, and 16 wk), while successful jumps from the perch to the floor increased with age (6.1, 7.5, 8.2, 8.9 at 4, 8, 13, and 16 wk, respectively). Failed landings decreased with age (<0.1 at 4, 8, and 16 wk, 0.0 at 13 wk). Additionally, failed jumps between perches decreased with age (0.1 at 4 wk, <0.1 at 8, 13, and 16 wk), and percent success increased with age (99.29%, 99.89%, 99.95%, 99.95% at 4, 8, 13, and 16 wk, respectively, Table 4). Overall, despite being numerically similar, total jumping accuracy increased with age (99.16%, 99.77%, 99.92%, 99.92% at 4, 8, 13, and 16 wk, respectively, Table 4).

### 3.3. Novel Object Test

There was no effect of L on the time taken to peck at the novel object (Table 6). LB pullets had a higher latency to peck (676 s) than LW pullets (212 s, *p* < 0.001, Table 6). There was no interaction between L and S.

### 3.4. H/L Ratio

There was no effect of L on H/L ratio (Table 7). LB pullets had a higher H/L ratio than LW pullets (0.26 vs. 0.13, respectively, *p* < 0.001, Table 7). There was no interaction between L and S.

## 4. Discussion

Behavioral observations are an important tool in assessing an animal’s response to its environment. To understand how S reacts differently to L at different ages, LB and LW pullets were reared to 16 wk. L did not influence the ability to identify behaviors; however S did have an impact. LB pullets’ behaviors were consistently more difficult to identify, regardless of L. This may have been due to the dark feather color of LB pullets that made it difficult to distinguish from the bedding when viewed in the infrared videos. Even though it was possible to identify the presence of an LB pullet, the challenge was identifying specifically what behavior the pullet was performing. Conversely, the white feather color of LW pullets provided a contrast against the litter and made for easier identification. Another challenge for identifying pullet behavior was their small size at 4 wk. A decrease in unidentified behavior with age was observed as the pullets’ body size increased.

It was hypothesized that pullet activity would increase with L; however, this was not observed. Rather, pullets reared at 10 or 30 lux spent more time jumping or flying than pullets reared at 50 lux at 4 wk, which was in contrast with previous literature that reported increased bird activity with L [6,15]. Interestingly, pullets reared at 50 lux were not occupied with pecking at objects in the environment, whereas previous studies reported increased visual stimulation at high L [24].

Across all recording periods, pullets reared at 10 lux spent more time pecking at the walls than pullets reared at 30 or 50 lux, while pullets reared at 30 and 50 lux were observed spending more time preening. Wall pecking is not a common behavior found or reported in other studies. However, the purpose of this behavior may be an extension of other exploratory behaviors. Kjaer and Vestergaard [25] suggested that low L may lead to a reduced ability to identify environmental cues and thus cause birds to increase the time spent exploratory pecking as compensation. Preening can be visually motivated [26], and higher L of 30 or 50 lux may encourage the pullets to maintain good plumage condition [24].

It is important to mention that there was no effect of L on injurious behavior, similar to a study by Hartini et al. [27] looking at 5 lux versus 60–80 lux. However, the results of this study is in contrast with Kjaer and Vestergaard [25], who reported two to three times more injurious pecking (reported as severe feather pecking in their paper) in pullets reared at 30 lux vs. 3 lux. This may be because of the type of light source used. The study by Kjaer and Vestergaard used incandescent light bulbs [25], while the present study used LED lights. Incandescent lights emit high amounts of red light, which were reported to increase injurious pecking activity [28,29]. The LED lighting used in this experiment was not red-saturated, and LED lights were reported to be preferred by chickens over incandescent lighting [30]. In the present study, feather condition was not measured. However, there was no obvious change in feather condition throughout the trial. Additionally, injurious pecking is multifactorial and is affected by strain, diet, and other environmental and management conditions [31].

The success of pullet jumps was high through all observation periods; however, it is important to note that jumping frequency increased with age and so did jumping accuracy. This supports the importance of preparing pullets for navigating a complex environment by exposing them to the same environment during the rearing period [1,32]. The jumps from the floor to the ramp were highest at 4 wk and decreased with age, highlighting the importance of providing ramps to facilitate movement between landing platforms and tiers [33]. L may also play a role in improving pullet vision for navigational jumps within the environment. Pullets reared at 30 lux performed more jumps than those reared at 10 lux. However, despite this, jumping accuracy was not affected by L, which was in agreeance with Moinard et al. [34], who studied jumping accuracy in hens reared at 5, 10, and 20 lux. Results from the present study suggest that 10 lux is bright enough for pullets to navigate their environment successfully.

Several studies reported increased fear and/or stress levels with increasing L due to increased injurious pecking [3,25]. Results from the present study reported no effect of L on fear or stress responses. This was in agreeance with behavior observations from this study, which reported minimal levels of injurious behavior. Possible explanations for disagreement between studies may be due to type of light source used, evenness of light distribution, and age of birds. However, based on the result of this study, L of 10 to 50 lux did not affect the fear or stress levels of pullets.

Several S differences were reported for behavioral observations, jumping frequency, and fear and stress responses. This may be explained by the characteristic differences between brown- and white-feathered birds. White-feathered pullets are more reactive and flightier than brown-feathered strains [10], which may explain why jumping frequency was higher in LW than LB pullets. LW pullets are also comparatively lighter than LB pullets and can easily generate enough energy to perform aerial ascents within their environment [34,35]. In comparison, LB pullets exhibit more proactive and exploratory characteristics [11,36], as evidenced by the increased time spent on the floor performing exploratory behaviors compared to LW pullets. These S differences could explain the fear and stress responses. LB pullets had a higher latency to peck at the novel object and had higher H/L ratios. Typically, longer latencies to peck at a novel object, or the higher the H/L ratio, are interpreted as indicators of more fear and stress [15,37]. However, LB pullets’ higher latency to peck at the object and higher H/L ratio may not be due to a higher fear and stress level, but rather due to different hormonal and behavioral responses to a stressor compared to LW pullets [11].

## 5. Conclusions

In conclusion, the results of the study suggest that light intensities of 10, 30, or 50 lux result in minor changes in behavior, with a small increase in preening at higher lux and a small increase in wall pecking at lower lux. Light intensity did not impact injurious behavior, fear, or stress levels of pullets up to 16 wk. All pullets increased their time spent preening as they aged. Total number of jumps and jumping accuracy increased with age, supporting the importance of rearing pullets in complex environments, especially if they will be housed in a similar environment during the laying phase. Light intensities above 30 lux may slightly increase jumping frequency; however, 10 lux is sufficient for pullets to jump within their environment successfully.

## Figures and Tables

**Table 1:** Ethogram for pullets, adapted from [18,19,20,21,22,23].

Behavior	Definition
Active behavior	
Standing	Body in upright and idle position [20]
Walking	Taking at least two successive steps [18]
Jumping or flying	Both feet in the air with wings flapping [21]
Resting behavior	Lying down or crouching with breast on floor or head tucked under wing, otherwise inactive [22]
Comfort behavior	
Preening	Manipulating own feathers with beak while standing or laying [20]
Wing or leg stretching	Extending wing or leg out to side or behind body and returning wing or leg back under body without taking a step forward [20]
Tail wagging	Moving tail side to side without moving rest of body [20]
Head shaking	Head moving side to side or up and down rapidly, body immobile [20]
Head scratching	Extending leg forward and upward to scratch head or neck [20]
Feather ruffling	Raising or shaking out feathers of wings and body [22]
Dustbathing	Rubbing body against floor and performing full body shake [22]
Wing flapping	Extending wings away from body and flapping up and down rapidly but without flight [20]
Nutritive behavior	
At the feeder	Standing or sitting with head extended into feeder [18]
At the drinker	Pecking at nipple drinker [22]
Exploratory behavior	
Gentle feather pecking	Pecking at other birds that does not cause harm or damage to plumage [20]
Wall pecking	Pecking at pen walls [20]
Object pecking	Pecking at perch, ramp, feeder tube (not feed pan), drinker (not nipples) [20]
Litter pecking	Pecking at straw or litter [20]
Ground scratching	Scratching movements on ground while crouching slightly [20]
Head sweeping	Rubbing beak from side to side [20]
Injurious behavior	
Injurious pecking	Pecking at other birds directed at head and neck but may include feet, causes recipient to flinch or escape environment [23]
Fighting	Sparring, leaping, wing flapping toward opponent and can include pecking [19]
Unidentified	Behavior unidentifiable, action of bird cannot be seen

**Table 2:** Average percentage of time (%) spent on each behavior by Lohmann Brown-Lite (LB) and Lohmann Selected Leghorn Lite (LW) pullets reared in floor pens under light intensities of 10, 30, or 50 lux over 12 h of light at 4 wk, and 8 h of light at 8, 13, and 16 wk of age.

	Light Intensity (L)				Strain (S)			Week of Age (wk)				
	10	30	50	*p*	LB	LW	*p*	4	8	13	16	*p*
Standing	23.6	23.5	23.3	0.94	23.2	23.7	0.68	20.6 ^c^	15.2 ^d^	25.5 ^b^	32.5 ^a^	<0.01
Walking	4.5	5.0	5.1	0.09	4.7	5.0	0.39	5.3 ^a^	5.4 ^a^	4.0 ^b^	4.9 ^a^	<0.01
Jumping or flying	0.1	0.1	0.2	0.34	0.1	0.1	0.67	0.1	0.2	0.1	0.1	0.10
Resting	13.5	14.2	13.9	0.46	10.8	17.0	<0.01	11.4 ^c^	21.0 ^a^	14.5 ^b^	8.7 ^d^	<0.01
Preening	10.1 ^b^	11.3 ^a^	12.1 ^a^	<0.01	9.8	12.6	<0.01	5.8 ^c^	11.9 ^b^	13.1 ^ab^	13.8 ^a^	<0.01
Comfort ^1^	1.0^b^	1.1 ^ab^	1.5 ^a^	0.01	1.1	1.2	0.17	0.9 ^b^	1.6 ^a^	1.5 ^a^	0.7 ^b^	<0.01
At the feeder	9.8	9.6	9.6	0.71	9.4	9.9	0.19	11.4 ^a^	10.6 ^a^	8.9 ^b^	7.7 ^c^	<0.01
At the drinker	3.4	3.3	3.4	0.46	3.3	3.4	0.12	3.0 ^b^	4.0 ^a^	3.4 ^b^	3.0 ^b^	<0.01
Gentle pecking	0.4	0.3	0.4	0.26	0.4	0.4	0.94	0.2 ^b^	0.4 ^a^	0.4 ^a^	0.5 ^a^	<0.01
Litter directed ^2^	17.4	17.3	17.5	0.95	19.7	15.2	<0.01	19.7 ^a^	17.4 ^b^	16.8 ^b^	15.9 ^b^	0.02
Wall pecking	3.8 ^a^	2.7 ^b^	2.3 ^b^	<0.01	3.6	2.2	<0.01	1.7 ^c^	2.7 ^bc^	3.3 ^ab^	4.0 ^a^	<0.01
Object pecking ^3^	0.5	0.4	0.5	0.62	0.5	0.5	1.00	0.2 ^c^	0.4 ^b^	0.5 ^ab^	0.9 ^a^	<0.01
Injurious ^4^	<0.1	<0.1	<0.1	0.06	<0.1	<0.1	0.70	<0.1	<0.1	<0.1	<0.1	0.82
Unidentified	11.8	11.3	11.0	0.66	13.6	9.1	<0.01	19.7 ^a^	10.1 ^b^	8.3 ^c^	7.5 ^c^	<0.01
*p* for Interactions			L × S		L × wk		S × wk		L × S × wk		SEM ^5^	
Standing			0.17		0.60		0.15		0.97		0.74	
Walking			0.52		0.59		0.77		0.62		0.12	
Jumping or flying			0.21		0.02 *		0.42		0.08		0.02	
Resting			0.39		0.77		0.47		0.98		0.65	
Preening			0.90		0.16		0.61		0.88		0.41	
Comfort ^1^			0.31		0.36		0.34		0.86		0.09	
At the feeder			0.92		0.49		0.03 *		0.98		0.29	
At the drinker			0.91		0.06		0.37		0.53		0.08	
Gentle pecking			0.41		0.39		<0.01 *		0.34		0.03	
Litter directed ^2^			0.97		0.44		<0.01 *		0.82		0.38	
Wall pecking			0.57		0.92		<0.01 *		0.58		0.21	
Object pecking ^3^			0.38		0.02*		0.03 *		0.95		0.05	
Injurious ^4^			0.32		0.79		0.86		0.48		0.01	
Unidentified			0.02 *		0.29		0.31		0.33		0.63	

^a–d^ Means within rows with different letters indicate a significant difference (*p* < 0.05). * Indicates a significant difference within interactions (*p* < 0.05). ^1^ Wing or leg stretching, tail wagging, head shaking, head scratching, feather ruffling, dustbathing, and wing flapping. ^2^ Behavior directed toward ground, including litter pecking, ground scratching, and head sweeping. ^3^ Pecking at perch, ramp, drinker, or feeder bin. ^4^ Injurious pecking and fighting. ^5^ SEM—Standard error of mean.

**Table 3:** Interactions between light intensity, strain, and week of age for behavioral expression of Lohmann Brown-Lite (LB) or Lohmann Selected Leghorn Lite (LW) pullets reared in floor pens under light intensities of 10, 30, or 50 lux.

		Week of Age (wk)			
		4	8	13	16
Percentage of Time (%) Spent on Each Behavior	Light Intensity (lux)				
Jumping or flying	10	0.11 ^ab^	0.11 ^abc^	0.02 ^bc^	0.07 ^bc^
	30	0.12 ^ab^	0.10 ^abc^	0.05 ^bc^	0.05 ^c^
	50	0.04 ^c^	0.42 ^a^	0.12 ^abc^	0.04 ^c^
Object pecking	10	0.29 ^e^	0.33 ^de^	0.61 ^abc^	0.85 ^ab^
	30	0.25 ^e^	0.41 ^bcde^	0.38 ^cde^	0.75 ^abcd^
	50	0.08 ^f^	0.34 ^de^	0.51 ^abcd^	1.06 ^a^
	**Strain**				
At the feeder	LB	10.60 ^ab^	10.62 ^a^	9.34 ^ab^	7.22 ^c^
	LW	12.24 ^a^	10.65 ^a^	8.43 ^bc^	8.20 ^bc^
Gentle pecking	LB	0.20 ^d^	0.18 ^cd^	0.37 ^bcd^	0.77 ^a^
	LW	0.22 ^d^	0.65 ^ab^	0.47 ^abc^	0.16 ^d^
Litter directed	LB	23.04 ^a^	20.97 ^ab^	18.58 ^bc^	16.10 ^d^
	LW	16.31 ^cd^	13.83 ^d^	15.06 ^d^	15.62 ^d^
Wall pecking	LB	1.41 ^b^	3.11 ^b^	4.43 ^a^	5.61 ^a^
	LW	1.99 ^b^	2.32 ^b^	2.22 ^b^	2.34 ^b^
Object pecking	LB	0.26 ^bc^	0.26 ^bc^	0.39 ^abc^	1.11 ^a^
	LW	0.16 ^c^	0.46 ^ab^	0.60 ^ab^	0.66 ^ab^
		**Light intensity (lux)**			
		10	30	50	
Unidentified	LB	14.77 ^a^	13.67 ^a^	12.34 ^a^	
	LW	8.83 ^b^	8.87 ^b^	9.71 ^b^	

^a–f^ Means within a behavior with different letters indicate a significant difference (*p* < 0.05).

**Table 4:** Average number of successful, failed, and percent success of jumps per bird directed upward, downward, and across by Lohmann Brown-Lite (LB) or Lohmann Selected Leghorn Lite (LW) pullets reared in floor pens under light intensities of 10, 30, or 50 lux over 24 h at 4, 8, 13, and 16 wk of age.

			Light Intensity (L)				Strain (S)			Week of Age (wk)				
From	To		10	30	50	*p*	LB	LW	*p*	4	8	13	16	*p*
Jumps upward														
Floor	Drinker	S	3.1	3.7	3.9	0.33	2.4	4.7	<0.01	4.7	3.2	3.1	3.2	0.19
		F	<0.1	<0.1	<0.1	0.71	<0.1	0.1	<0.01	0.1 ^a^	<0.1 ^b^	<0.1 ^bc^	<0.1 ^c^	<0.01
		%	98.44	99.22	99.42	0.19	99.20	98.85	0.47	97.83 ^b^	98.77 ^ab^	99.66 ^a^	99.85 ^a^	0.01
Floor	Ramp	S	0.4	0.4	0.6	0.22	0.3	0.6	0.04	1.3 ^a^	0.4 ^b^	0.2 ^c^	0.1 ^d^	<0.01
		F	<0.1	<0.1	<0.1	0.78	<0.1 ^b^	<0.1 ^a^	0.03	<0.1 ^a^	<0.1 ^b^	<0.1 ^b^	0.0 ^b^	<0.01
		%	98.71	99.50	99.82	0.35	99.12	99.59	0.40	98.74	99.74	98.96	100.00	0.44
Floor	Perch	S	9.0	9.3	9.3	0.70	4.9	13.5	<0.01	4.9 ^b^	10.4 ^a^	10.9 ^a^	10.7 ^a^	<0.01
		F	<0.1	<0.1	<0.1	0.24	<0.1 ^b^	<0.1 ^a^	<0.01	0.1 ^a^	<0.1 ^a^	<0.1 ^b^	<0.1 ^b^	<0.01
		%	99.07	99.12	99.20	0.95	98.64	99.61	0.01	97.15 ^c^	99.64 ^b^	99.89 ^ab^	99.83 ^a^	<0.01
Jumps downward														
Drinker	Floor	S	3.0	3.5	3.7	0.36	2.4	4.5	<0.01	4.6	3.0	2.9	3.2	0.06
		F	0.0	<0.1	<0.1	0.30	0.0	<0.1	0.03	<0.1	0.0	0.0	<0.1	0.07
		%	100.00	99.98	99.98	0.33	100.00	99.97	0.04	99.96	100.00	100.00	99.98	0.14
Ramp	Floor	S	0.2	0.2	0.2	0.42	0.2	0.2	0.09	0.4 ^a^	0.1 ^b^	0.1 ^b^	0.1 ^b^	<0.01
		F	0.0	0.0	0.0	-	0.0	0.0	-	0.0	0.0	0.0	0.0	-
		%	100.00	100.00	100.00	-	100.00	100.00	-	100.00	100.00	100.00	100.00	-
Perch	Floor	S	7.5	7.6	7.9	0.71	3.5	11.8	<0.01	6.1 ^c^	7.5 ^b^	8.2 ^b^	8.9 ^a^	<0.01
		F	<0.1	<0.1	<0.1	0.47	<0.1	<0.1	0.95	<0.1 ^a^	<0.1 ^ab^	0.0 ^b^	<0.1 ^ab^	0.04
		%	99.98	99.96	99.96	0.80	99.94	99.98	0.13	99.87 ^b^	99.99 ^a^	100.00 ^a^	99.99 ^a^	<0.01
Jumps across														
Perch	Perch	S	6.9 ^b^	8.3 ^a^	7.6 ^ab^	0.04	7.8	7.4	0.35	7.2 ^b^	9.5 ^a^	6.9 ^b^	6.9 ^b^	<0.01
		F	<0.1	<0.1	<0.1	0.33	<0.1	<0.1	0.56	0.1 ^a^	<0.1 ^b^	<0.1 ^c^	<0.1 ^bc^	<0.01
		%	99.71	99.75	99.85	0.38	99.79	99.75	0.71	99.29 ^b^	99.89 ^a^	99.95 ^a^	99.95 ^a^	<0.01
Total														
		S	30.1	33.1	33.2	0.31	21.5	42.8	<0.01	29.2 ^b^	34.0 ^a^	32.2 ^a^	33.1 ^a^	<0.01
		F	0.1	0.1	0.1	0.42	0.1 ^b^	0.1 ^a^	<0.01	0.2 ^a^	0.1 ^b^	<0.1 ^c^	<0.1 ^c^	<0.01
		%	99.66	99.69	99.73	0.59	99.68	99.70	0.65	99.16 ^c^	99.77 ^b^	99.92 ^a^	99.92 ^a^	<0.01
*p* for Interactions				L × S			L × wk		S × wk		L × S × wk		SEM ^1^	
Jumps upward														
Floor	Drinker	S		0.30			0.99		<0.01 *		0.73		0.23	
		F		0.75			0.64		0.01 *		0.93		0.01	
		%		0.78			0.23		0.80		0.58		0.22	
Floor	Ramp	S		0.91			0.30		0.01 *		0.52		0.07	
		F		0.50			0.84		<0.01 *		0.93		<0.01	
		%		0.15			0.58		0.46		0.71		0.30	
Floor	Perch	S		0.71			0.54		<0.01 *		0.64		0.55	
		F		0.51			0.77		0.28		0.84		<0.01	
		%		0.70			0.79		0.04 *		0.91		0.21	
Jumps downward														
Drinker	Floor	S		0.31			0.99		<0.01 *		0.78		0.22	
		F		0.30			0.29		0.07		0.29		<0.01	
		%		0.33			0.16		0.14		0.16		0.01	
Ramp	Floor	S		0.33			0.53		0.89		0.18		0.02	
		F		-			-		-		-		0.00	
		%		-			-		-		-		0.00	
Perch	Floor	S		0.95			0.97		0.39		0.84		0.46	
		F		<0.01 *			0.37		0.03 *		0.38		<0.01	
		%		0.07			0.43		0.01 *		0.24		0.02	
Jumps across														
Perch	Perch	S		0.74			0.79		<0.01 *		0.97		0.22	
		F		0.73			0.49		0.86		0.94		<0.01	
		%		0.73			0.50		0.73		0.97		0.05	
Total														
		S		0.76			0.97		<0.01 *		0.99		1.25	
		F		0.45			0.71		0.01 *		0.97		0.01	
		%		0.44			0.44		0.76		0.85		0.04	

S—Success. F—Failure. %—Percent success. ^a–c^ Means within a row with different letters indicate a significant difference (*p* < 0.05). * Indicates a significant difference within interactions (*p* < 0.05). ^1^ SEM—Standard error of mean.

**Table 5:** Interactions between light intensity, strain, and week of age for jumping frequency and success of Lohmann Brown-Lite (LB) or Lohmann Selected Leghorn Lite (LW) pullets reared in floor pens under light intensities of 10, 30, or 50 lux.

Average Jumps Per Pullet over 24 h				Week of Age (wk)			
				4	8	13	16
Upward			**Strain**				
Floor	Drinker	S	LB	1.62 ^c^	3.04 ^b^	2.43 ^bc^	2.57 ^bc^
			LW	7.75 ^a^	3.39 ^b^	3.68 ^b^	3.71 ^b^
		F	LB	0.01 ^bc^	0.02 ^bc^	0.01 ^bc^	<0.01 ^c^
			LW	0.17 ^a^	0.03 ^b^	0.01 ^bc^	0.01 ^bc^
Floor	Ramp	S	LB	0.78 ^ab^	0.36 ^bc^	0.14 ^d^	0.05 ^e^
			LW	1.77 ^a^	0.36 ^b^	0.17 ^cd^	0.12 ^de^
		F	LB	0.01 ^b^	0.00 ^b^	<0.01 ^b^	0.00 ^b^
			LW	0.03 ^a^	<0.01 ^b^	0.00 ^b^	0.00 ^b^
Floor	Perch	S	LB	1.15 ^e^	5.49 ^d^	6.78 ^bc^	6.25 ^cd^
			LW	8.59 ^b^	15.22 ^a^	15.00 ^a^	15.16 ^a^
		%	LB	95.18 ^b^	99.68 ^a^	99.90 ^a^	99.81 ^a^
			LW	99.12 ^ab^	99.61 ^a^	99.88 ^a^	99.85 ^a^
Downward							
Drinker	Floor	S	LB	1.57 ^c^	2.94 ^b^	2.37 ^bc^	2.55 ^bc^
			LW	7.62 ^a^	3.01 ^b^	3.46 ^b^	3.84 ^b^
		F	LB	0.01 ^a^	0.00 ^b^	0.00 ^b^	0.00 ^b^
			LW	<0.01 ^ab^	<0.01 ^ab^	0.00 ^b^	<0.01 ^ab^
Perch	Floor	%	LB	99.77 ^b^	100.00 ^a^	100.00 ^a^	100.00 ^a^
			LW	99.98 ^a^	99.99 ^a^	100.00 ^a^	99.97 ^a^
Across							
Perch	Perch	S	LB	6.39 ^cd^	8.98 ^ab^	7.79 ^bc^	8.04 ^abc^
			LW	8.02 ^abcd^	9.97 ^a^	5.92 ^d^	5.77 ^d^
Total		S	LB	14.40 ^c^	24.29 ^b^	23.46 ^b^	23.86 ^b^
			LW	43.94 ^a^	43.66 ^a^	41.03 ^a^	42.41 ^a^
		F	LB	0.12 ^bc^	0.05 ^cd^	0.02 ^d^	0.02 ^d^
			LW	0.33 ^a^	0.10 ^b^	0.04 ^cd^	0.04 ^cd^
				**Light Intensity (lux)**			
Downward				10	30	50	
Perch	Floor	F	LB	0.00 ^b^	0.002 ^ab^	0.003 ^ab^	
			LW	0.01 ^a^	0.00 ^b^	0.00 ^b^	

S—Success. F—Failure. %—Percent success. ^a–e^ Means within a successful or failed landing with different letters indicate a significant difference (*p* < 0.05).

**Table 6:** Latency to peck at novel object (seconds) by Lohmann Brown-Lite (LB) or Lohmann Selected Leghorn Lite (LW) pullets reared in floor pens under light intensities of 10, 30, or 50 lux at 15 wk of age (8 pen replicates per L × S).

Light Intensity (L)				Strain (S)			L × S	
10	30	50	*p*	LB	LW	*p*	*p*	SEM ^1^
397	497	437	0.436	676	212	<0.001	0.415	41.9

^1^ SEM—Standard error of mean.

**Table 7:** Heterophil/lymphocyte ratios of Lohmann Brown-Lite (LB) and Lohmann Selected Leghorn Lite (LW) pullets reared in floor pens under light intensities of 10, 30, or 50 lux at 15 wk of age (12 pen replicates per L × S).

Light Intensity (L)				Strain (S)			L × S	
10	30	50	*p*	LB	LW	*p*	*p*	SEM ^1^
0.20	0.18	0.20	0.507	0.26	0.13	<0.001	0.922	0.011

^1^ SEM—Standard error of mean.

## Data Availability

The datasets used and/or analyzed during the current study are available from the corresponding author upon reasonable request.
